# Sand Mandala: A Case Study in the Essence of Psychological Resilience, Growth, and Thriving in Life After Stroke

**DOI:** 10.3390/healthcare14101370

**Published:** 2026-05-16

**Authors:** Erin Doan, Lori Gray, Heather Noble, Julie Bertram

**Affiliations:** 1 Saint Luke’s Neurological Consultants, Saint Luke’s Marion Bloch Neuroscience Institute, BJC Saint Luke’s Health System, Kansas City, MO 64109, USA; etompkins@saintlukeskc.org; 2 Holistic Health and Contemplative Well-Being, School of Interdisciplinary Health Programs, Western Michigan University, Kalamazoo, MI 49008, USA; 3 Independent Contractor, Prairie Village, KS 66208, USA; hnoble2@kumc.edu; 4 College of Nursing, University of Missouri-St. Louis, St. Louis, MO 63121, USA; bertramje@umsystem.edu

**Keywords:** mindfulness, mindfulness-based stress reduction, psychological resilience, stroke, grounded theory

## Abstract

**Highlights:**

This qualitative instrumental case study examines how one stroke survivor and his carepartner described long-term recovery after participation in the Mindfulness-Based Recovery from Stroke program.

**What are the main findings?**

- Three interrelated themes described resilience in this case: antecedents to poststroke resilience, the obstacle is the way to posttraumatic growth, and poststroke resilience as mindfulness embodied.
- The findings suggest that prestroke strengths, responses to adversity, and mindfulness practice may have interacted in shaping this participant’s long-term recovery.

**What are the implications of the main findings?**

- This case offers an exploratory, process-oriented account of how resilience and mindfulness may interact after stroke.
- The findings may inform future research on long-term, whole-person approaches to stroke rehabilitation in larger and more diverse populations.

**Abstract:**

**Background/Objectives:** Psychological resilience is associated with improved quality of life after stroke, yet less is known about how resilience and mindfulness interact over time in long-term recovery. This qualitative instrumental case study aimed to describe how psychological resilience and mindfulness unfolded in one stroke survivor with high psychological resilience and to identify processes that may inform future research on long-term stroke rehabilitation. **Method:** We conducted an instrumental qualitative case study involving a stroke survivor and his spouse (carepartner), using an in-depth interview framework for data collection and grounded theory procedures to support analysis. Data sources included interview transcripts and contextual artifacts, such as personal and professional websites, emails, and informal conversations. **Findings:** Three interrelated themes described how resilience unfolded in this case: (a) antecedents to poststroke resilience: personality traits, values, behavioral modeling, and environmental factors; (b) the obstacle is the way to posttraumatic growth; and (c) poststroke resilience: mindfulness embodied. Together, these themes suggest how prestroke strengths, responses to adversity, and mindfulness practice interacted in this participant’s long-term recovery. **Conclusions:** This exploratory case study offers a detailed account of how resilience and mindfulness may interact after stroke in one highly resilient individual. The findings are hypothesis-generating and may help guide future research on mindfulness-based and holistic approaches to long-term stroke recovery in larger and more diverse populations.

## 1. Introduction

Psychological resilience allows individuals to successfully cope with adversity such as living with chronic stroke. An essential indicator of psychosocial function and quality of life in chronic disease, psychological resilience equips individuals with chronic disease to cope with challenges and protects them against common comorbid health conditions such as depression, shifting the focus of stroke recovery from avoidance of mental illness to promotion of health [1]. Psychological resilience is independently associated with positive chronic stroke outcomes, such as cognitive outcomes involving mood, memory, and communication [2], and has a mediating effect on sleep disturbance and poststroke depression [3]. However, despite evidence that psychological resilience is helpful in chronic disease such as stroke, few studies have focused on improving psychological resilience in chronic stroke. One possible pathway to build psychological resilience in chronic stroke is formal mindfulness meditation training and practice. We conducted a pilot study of an 8-week curriculum for people who have experienced stroke that uses mindfulness-based practices to promote recovery from stroke [4], which was in turn based on the conceptual model of Mindfulness-Based Recovery from Stroke [5]. The pilot study provided support that psychological resilience is beneficial to the survivor’s emotional recovery from stroke and increases their likelihood of long-term personal investment in the ongoing journey of stroke recovery.

Although prior research suggests that psychological resilience is associated with better emotional and cognitive outcomes after stroke, less is known about how resilience is experienced, sustained, and shaped over time in individual stroke survivors, particularly in relation to mindfulness-based intervention and long-term recovery. Existing studies provide important information on outcomes, but fewer have examined the lived processes through which resilience, meaning-making, and mindfulness may interact in depth within a single case. An instrumental case study can help illuminate these processes and generate hypotheses for future research.

In this article, we conceptualize psychological resilience in chronic stroke as a dynamic process by which individuals resist, recover from, and rebound after adversity, attaining equal or higher levels of functioning across emotional, cognitive, behavioral, and relational domains. Drawing on the society-to-cells resilience theory, we locate this process within interacting levels of influence—societal, community, family, individual, trait, and cellular—and attend to how these factors shape resilience over time. In keeping with posttraumatic growth theory and Stoic philosophy, we also understand resilience as encompassing positive psychological changes, such as transformed personal values, greater appreciation of life, openness to new possibilities, and spiritual development, that emerge in response to the trauma of stroke.

Accordingly, our objective in this instrumental case study was to trace how psychological resilience manifested and evolved in one stroke survivor with exceptional resilience as he engaged in the Mindfulness-Based Recovery from Stroke (MBRfS) program and navigated life after stroke. To examine this process, we focused on how the participant and his carepartner described experiences, coping strategies, values, and relationships over time and how these narratives reflected the defining attributes and components of psychological resilience. Guided by the society-to-cells resilience theory, Stoic philosophy, and the posttraumatic growth framework, we treated psychological resilience as a central sensitizing concept for our interview guide and for our grounded theory analysis.

This article presents an in-depth case study of one participant with exceptional psychological resilience from the pilot study, with whom we conducted additional interviews and analyses 10 months after the completion of MBRfS. We selected this participant as an instrumental and extreme case because highly resilient trajectories can make adaptive processes more visible and conceptually accessible. Our purpose was not to claim representativeness but to use this case to deepen understanding of resilience-related processes and generate hypotheses for future study. Our intention is not to claim that his trajectory represents a typical outcome of mindfulness-based interventions after stroke but rather to illuminate how psychological resilience and mindfulness may interact in one extreme case and to generate hypotheses for future research on long-term, whole-person stroke rehabilitation.

We met our participant, “Robert” (pseudonym), during the first cycle of the pilot study [4], when he appeared to embody several components of psychological resilience. He remained engaged with rehabilitation activities, positive thinking, and goal setting across follow-up encounters. He reported social support, hope for the future, acceptance, flexibility, discipline, and a desire to find meaning in the stroke experience. We selected Robert as an extreme case of high psychological resilience because his trajectory offered an opportunity to examine how resilience and mindfulness may interact in long-term recovery. In his own words, Robert stated, “I want to find something with this stroke that is going to impact my life positively.” This case was therefore used to explore resilience-related processes in depth, rather than to represent a typical outcome after stroke or mindfulness-based intervention.

## 2. Materials and Methods

### 2.1. Study Design and Theoretical Framework

To examine psychological resilience in the setting of ischemic stroke, we designed a bounded qualitative instrumental case study with a grounded theory analysis [6]. We developed this case study of Robert in part to build on an emerging grounded theory of Mindfulness-Based Recovery from Stroke [5], which informed the design of the pilot study in which we met Robert [4]. We used Seidman’s [7] in-depth interview framework to guide data collection and grounded theory procedures, including open, axial, and selective coding, to support analysis of the case.

We led with the assumption that exceptional psychological resilience, as exemplified in this case study, is dynamic and modifiable, a quality or state within and surrounding a person after stroke that serves as a buffer to emotional changes and galvanizes optimal stroke recovery. In keeping with our conceptualization in the introduction, we approached psychological resilience as a process that unfolds across emotional, cognitive, behavioral, and relational domains rather than as a fixed trait. We were guided by several theoretical frameworks, including the previously mentioned Mindfulness-Based Recovery from Stroke [5] as well as the society-to-cells resilience theory [8], Stoic philosophy [9,10], and the posttraumatic growth framework [11]. These frameworks served as sensitizing concepts to support interpretation rather than as fixed deductive coding structures, and we remained attentive to patterns and meanings that emerged inductively from the data.

We used the society-to-cells resilience theory as a framework to create the interview structure and question domains [8]. This theory emphasizes the potential for psychological resilience in all people and how it is affected by the dynamic interaction of factors at six levels: society, community, family, individual, trait, and cellular. It views psychological resilience multidimensionally and acknowledges how groups with diverse racial, ethnic, socioeconomic, gender, and other characteristics experience health and social inequities. It also incorporates social determinants of health by considering outcomes at levels such as community and societal structures, family behavior modeling, coping strategies, brain function, and oxidative stress.

We used the society-to-cells resilience theory as a framework to structure both data collection and analysis. The semistructured interview guides were organized around factors at the societal, community, family, individual, trait, and cellular levels, including prompts about life history, social roles, social determinants of health, health behaviors, coping strategies, and perceived sources of vulnerability and strength. During the interviews, we invited Robert and his carepartner to describe challenges and resources before and after stroke, turning points in his recovery, and the influence of the Mindfulness-Based Recovery from Stroke program on his capacity to meet adversity. This design allowed us to elicit rich descriptions of how resilience was experienced, supported, and tested over time.

In the analytic phase, we treated psychological resilience as the central sensitizing concept guiding coding and category development. During open coding, we labeled segments of text in relation to resilience-relevant processes (e.g., discipline, courage, acceptance, spiritual growth, poststroke anger, community support, and embodied mindfulness). Through axial and selective coding, we then grouped these codes into broader categories that represent antecedents to resilience, ways of working with obstacles and posttraumatic growth, and mindfulness-embodied resilience after stroke.

Throughout the analytic phase, we also studied ancient philosophical tenets of Stoicism to fully understand the other nuanced components of psychological resilience [9,10]. Broadly, Stoicism is rooted in an understanding of order and personal ethics. It suggests that one can cultivate inner strength in any circumstance through both reason and virtue. Stoicism offers a principled, intellectual way of approaching challenges. Essentially, our participant, like the ancient Stoics, focuses on controlling not the world but how he responds to the world [12,13,14]. A Stoic approach allows Robert to live with courage, discipline, justice, and wisdom in every circumstance, whether large or small.

During the analytic process, we also adopted the posttraumatic growth framework as a guide to finding meaning in the data. Referring to the ancient idea that one can triumph over adversity, even if it leaves figurative or literal scars, posttraumatic growth emerged as a research concept in the mid-1990s, with more recent refinements [11]. This framework states that positive psychological changes can be the result of one’s struggle with trauma or a highly difficult situation such as recovery from stroke. Posttraumatic growth encompasses five domains: personal strength, greater appreciation of life, openness to new possibilities, spiritual development, and closer personal relationships. Throughout conversations regarding the analytic process, it became evident Robert manifested the personal growth and transformation central to these domains.

Thus, this study aimed to examine how psychological resilience and mindfulness interacted over time in one instrumental case of long-term stroke recovery. A secondary aim was to identify processes and contextual factors that may help explain resilience in this case and inform future research on holistic stroke rehabilitation.

### 2.2. Mindfulness-Based Recovery from Stroke Program

The Mindfulness-Based Recovery from Stroke (MBRfS) program is an 8-week, group-based mindfulness intervention adapted from Mindfulness-Based Stress Reduction for people living with stroke. Each weekly 2-h session included guided mindfulness practices (such as body scan, breath-focused sitting meditation, and gentle mindful movement adapted for stroke-related limitations), brief didactic teaching, and facilitated group discussion. The curriculum emphasized themes relevant to long-term stroke recovery, including accepting the stroke event and acquired brain injury, navigating uncertainty with awareness, listening to the body as a teacher, and cultivating self-compassion, patience, and nonjudgment toward changing abilities.

Participants were encouraged to engage in home practice between sessions (approximately 20–30 min per day) using audio recordings of guided practices and written reminders. Home practice emphasized brief, flexible exercises that could be integrated into daily routines and tailored to fatigue, aphasia, or motor limitations. In keeping with the MBRfS conceptual model, the program explicitly addressed cognitive, emotional, relational, and existential aspects of life after stroke—for example, exercises in observing thoughts related to fear of recurrence, mindful communication with carepartners, and reflecting on sources of meaning and hope. In this case, we were particularly interested in how these program components intersected with Robert’s preexisting resilience and contributed to his evolving narrative of recovery.

### 2.3. Procedure

Robert participated in our case study 18 months following a left middle cerebral artery embolic ischemic stroke and 10 months following his participation in the mindfulness-based pilot study [4]. We conducted three semistructured interviews with Robert over 3 weeks and a fourth interview with Robert’s carepartner (his wife) the following week.

We conducted the interviews following Seidman’s [7] in-depth three-stage interviewing framework. The first interview establishes the context of the participant’s experience and life history by asking them to tell as much as possible about themselves while considering the topic (here, recovery from stroke). The second interview allows the participant to reconstruct the details of their experience within the context in which it occurred. The third interview encourages the participant to reflect on the meaning of their experience, in this case, following the completion of the Mindfulness-Based Recovery from Stroke pilot. The fourth interview, with the carepartner, elicits input about the carepartner’s experience and explores their actions and observations. The carepartner interview also led us to ponder the ethics and responsibility of the caregivers of those affected by stroke. At the conclusion, we triangulated data from the first three interviews.

### 2.4. Ethical Considerations

The institutional review board (IRB) approved this substudy of the mindfulness-based pilot with an amendment to the original research proposal. Inclusion criteria for the pilot study included history of chronic ischemic stroke; English speaking; and the ability to communicate, comprehend, and answer open-ended questions. Exclusion criteria included end-stage disease of any kind, severe aphasia, active addictions, or uncontrolled mental and physical illness. The biggest and most significant risks of the mindfulness-based pilot study as shared with the participants were loss of privacy and emotional discomfort with reliving difficult times. To reduce these risks, we employed strategies such as maintaining meticulous deidentified data, transferring data by secure measures, and sending an outline of questions 24 h ahead of interviews. The participants were reminded at different intervals that participating in research is voluntary and that they may terminate their participation at any time.

We selected Robert’s case as an exemplar because he manifested qualities of exceptional psychological resilience. We contacted Robert and his wife first by email only and second by email and phone. After two discussions separated by time and space, a nurse stroke clinician on our team obtained informed consent. The nurse clinician researcher and psychologist researcher on our team conducted the four interviews via the Microsoft Teams platform to allow for direct observation by a third researcher, using a handheld digital recorder as a backup recording method. The nurse clinician researcher has more than 20 years of nursing experience, including 13 years of advanced practice nursing with a focus on stroke care. She was the principal investigator on the mindfulness-based pilot study. The psychologist researcher is a mindfulness-based teacher, tenured faculty member, and stroke survivor. She designed and led the mindfulness-based pilot 8-week class experience.

Each interview lasted approximately 1 h. Robert and his carepartner were in their home, and the researchers were located in their respective professional settings on secure and IRB-approved networks. Following the interviews, we reviewed artifacts of personal and professional websites, emails, and informal conversations. These materials were used as contextual and triangulating artifacts rather than as equivalent primary data sources. We included only materials that directly informed understanding of the participant’s recovery narrative or were public/provided accounts relevant to the case, and they were used to enrich context rather than to replace interview data. Microsoft Teams provided transcripts of the conversations, and other information was transcribed in Microsoft Word. We reviewed recordings and edited them by hand for a total of four transcripts.

Because members of the research team were involved in the pilot intervention and the broader stroke care context, we recognized the potential for interpretive bias. We addressed this through reflexive discussion throughout data collection and analysis, explicitly considering how our clinical, mindfulness, and theoretical commitments might shape interpretation. Team-based analytic review and triangulation across data sources were used to strengthen credibility and challenge our assumptions.

### 2.5. Data Analysis

The steps for data analysis of the bounded case study included open coding, axial coding, and selective coding, as recommended by Corbin and Strauss [15] and Strauss and Corbin [16]. The nurse, clinician, researcher, and psychologist conducted the initial coding, with ongoing review by the broader analytic team, including a qualitative grounded theory expert. Early transcripts were coded and discussed collaboratively to refine code definitions and develop a shared analytic structure. Through repeated comparison across interviews, memoing, and team discussion, initial codes were refined into broader categories, and interpretive differences were resolved through consensus rather than formal statistical reliability procedures.

The four axial codes developed initially from the first interview were (a) resilient personality traits; (b) childhood influences and personal values; (c) continuous sources of support; and (d) foundational components of physical, mental, and spiritual health. These codes clearly described the experience of the participant’s bounded case through the lens of the theoretical perspective of the society-to-cells resilience theory [8]. At the cellular and physiological level, Robert experienced potential inherent risks to his overall threat to psychological resilience based on all six levels of the society-to-cells resilience theory, including age, personal history of stress related to the life transition of retirement, selling his business, history of pulmonary embolism, and tobacco and alcohol consumption. These were offset by his maintenance of a healthy diet, exercise, and sleep; managing stress; and keeping a healthy weight. At the individual level, Robert had intact coping mechanisms and a positive sense of self. He was connected with his family and reported feeling protected within his family throughout the interviews. On the societal level, he had aspects of male gender and adequate education. Due to stroke-related challenges and social perspectives surrounding age, his career directions felt ambiguous. He described having navigated other challenging transitions throughout his life, including a divorce.

In addition to developing the three core categories, we undertook a focused review of the transcripts to identify discourse-level indices of mindfulness in Robert’s speech. Guided by contemporary descriptions of mindfulness, we attended to language that reflected present-moment awareness, observing thoughts, nonjudgmental acceptance, nonreactivity, compassion, and nonstriving. We then examined how and in what contexts these mindfulness-related ways of speaking appeared across the three participant interviews and the carepartner interview.

Our goal in this focused review was to characterize how mindfulness-related processes were woven into Robert’s evolving narrative of recovery, rather than to generate quantitative estimates of their occurrence. Given the single-case, instrumental design and the exploratory, theory-driven aims of this study, we did not undertake a formal quantitative frequency analysis of codes; such counts could suggest a level of measurement precision and generalizability that exceeds the scope of this in-depth case.

## 3. Findings

### 3.1. Axial Coding Evolution

An analytic team including a qualitative grounded theory expert abstracted and reorganized the analysis into three categories: (a) antecedents: personality traits, values, behavioral modeling, and environmental factors; (b) coping factors; and (c) poststroke resilience. Categories in grounded theory are composed of central concepts that emerge from the data and represent the phenomena being studied. They are the building blocks of the theory and help to organize and explain the data. The process of comparing concepts and categories leads to redefining them as more data are analyzed [17]. Subcategories are more specific elements within a category that further refine and expand meaning of the broader category [18]. Properties are the attributes or characteristics that define a category or subcategory; they help to describe and distinguish the specific features of the category or subcategory. Dimensions refer to the variation of properties along a continuum. They provide additional context and detail, helping to elaborate on the meaning of the category by offering various angles, viewpoints, and ranges [19].

Initial axial codes were revised repeatedly as interviews were compared across time and against contextual materials. Codes related to traits, values, and environmental supports were grouped into antecedents to poststroke resilience; codes related to struggle, adaptation, and reframing were integrated into “the obstacle is the way”; and codes related to awareness, acceptance, and nonstriving were consolidated into poststroke resilience as mindfulness embodied. This iterative process helped distinguish between prestroke foundations, responses to adversity, and mindfulness-related processes in the final analytic structure.

Through multiple engagements with the codes, concepts, and categories and a back-and-forth process with theory, literature, and data, we arrived at Category 1: antecedents to poststroke resilience: personality traits, values, behavioral modeling, and environmental factors; Category 2: the obstacle is the way to posttraumatic growth; and Category 3: poststroke resilience: mindfulness embodied.

### 3.2. Category 1: Antecedents to Poststroke Resilience: Personality Traits, Values, Behavioral Modeling, and Environmental Factors

Category 1, antecedents to poststroke resilience: personality traits, values, behavioral modeling, and environmental factors, had initial subcategories of personality attributes that led to the participant’s exceptional psychological resilience, such as being hard-working, steadfast, goal-oriented, driven, and physically active. The participant’s responses showed his curiosity often related to athletic hobbies. He stated in Interview 1,

[I like] sports like running, cycling, climbing, hiking…snowboarding…I like rehabbing buildings…I have land…all kinds of equipment and machinery like tractors that I can play with…fishing…being active and doing stuff…love reading…. There is a lot out there that interests me.

In Interview 2, when the researcher remarked on his artistic website, he responded, “There’s a lot out there if you stick your nose in it.” After completion of the three interviews, the codes continued to shift. In Interview 2, the code of “courage is my mantra” was prominent: “I’m going to make a full recovery, and I’m going to dive in. So, courage became my mantra.” Robert’s best friend’s mother had had a stroke, and hearing stories of her poststroke changes was motivating to his recovery:

He said that she recovered. He said she never recovered in her mind. That it just knocked her out and she didn’t want to communicate with the people…she just disconnected because she felt like she wasn’t what she once was. She just checked out…I’m not going to let that happen to me.

He frequently exhibited personal responsibility, beginning in Interview 1: “Life is an opportunity, and if you waste it, that is your fault. There is a lot to do out there; don’t waste any time.” This code in particular touches on the Stoic idea of universe transformation and that all things are impermanent [20].

Robert was driven by discipline, a primary Stoic virtue coded as it is not enough to do; be the best. In Interview 1, he stated,

It wasn’t enough to create a simple business to run. No, [in my mind, I thought,] “Let’s become the biggest. The best.” Or, [for another example,] when I [decide to] go for a jog, [it’s] not going to just jog…. Let’s enter a marathon and then run Boston.

This excerpt shows Robert’s strong drive and extreme expectations of himself that predated the stroke.

Behavioral modeling was seen in how Robert adapted his work habits, occupation, and hobbies. He noted many similarities to his father’s work life and his grandfather’s artistic side. In Interview 1, he demonstrated how highly he valued hard work. His father was a role model: “[He] had an amazing memory…and [was] a very hard worker. He would become my role model.” He also stated that being around his grandfather was “instrumental in rounding my personality. You know I wasn’t an automaton that was just driven for success” (Interview 1). In Interview 2, he remarked,

Dedication and perseverance will prevail. That’s how I have done everything that means a great deal to me. I’ve had this belief that anything that’s really worthwhile requires a struggle. The things that are dearest to me are also the things that I had to work the hardest for, and the lessons that I’ve learned in life didn’t come from successes. That came from failures. That’s kind of the cost of education.

This outlook of “the cost of education” the Stoics refer to as the “tax of life” and demonstrates the virtue of wisdom. Stoic philosopher Seneca wrote to Lucilius, “All the things which cause complaint or dread are like the taxes of life—things from which, my dear Lucilius, you should never hope for exemption or seek escape” [21] (Letter 96, Section 2).

Environmental factors are organized into this category of antecedents to poststroke resilience. For example, Robert left home at 16 and learned the benefit of education at prep school out of state: “It was an incredible experience. It allowed me to catch up on the many years I was avoiding school.” He has access to healthy, primarily plant-based foods and spends time riding his bike outdoors to experience fresh air. As part of stress management discussed in Interview 1, he stated, “I do like getting out in nature. You know just to be part of it.” He takes care of land in the country, chops wood, and removes trees.

Robert values community, sharing, and hearing stories. He shared the prominent impact of belonging to like-minded groups, such as the mindfulness-based group or Alcoholics Anonymous (AA). Sharing stories leads to understanding, compassion for self and others, and decreased feelings of isolation. During Interview 3, Robert stated,

Sharing amongst everybody is really valuable. That is another AA-type thing to hear people talk about their struggles, their failures, their hopes and dreams and successes. All the good and the bad you can share with the community, everyone would benefit.

### 3.3. Category 2: The Obstacle Is the Way to Posttraumatic Growth

The second category of coping factors was expanded transiently to barriers and facilitators of resilience, and in its last iteration, the obstacle is the way to posttraumatic growth. During the analysis of Interview 2, coping factors were included as facilitators to manage Robert’s physical, mental, and spiritual health. Additionally, he had some negative coping factors, such as prior alcoholism and tobacco dependence. Initially, codes were seen as slowly adding or taking away from psychological resilience. He described the stroke as “misery” and “agonizing” but was also able to express gratitude for the ability to move his body: “The left side was not responding as well. I was so blessed to just still be able to move. I could run, I could cycle” (Interview 2). Gratitude and acceptance describe how Robert was coping with the situation and embracing the Stoic law of reason: Humans cannot escape the universal law of reason, but they can deliberately align with it through acceptance and mindful choices [20,22]. We noted further positive coping, the power of encouragement from medical staff, and a growth mindset:

It took me about 2 days to say, “Oh alright this is the situation we’ve been dealt with, and we’ve got to embrace this because there’s only one way out. They say I can get better and so I’m going to go after it…just like everything else I do.”

Robert described challenges since the stroke: “I think my challenges are the stroke. It caused me to be fearful of stuff. I think it’s because the event was so traumatic” (Interview 2). This was a barrier to having courage at times during his recovery. Also in Interview 2, he described a personal challenge leading to an interpersonal issue:

I have a little more, I’ve more challenges in handling my emotions. So, I can get frustrated much easier early on quite a bit the first 6 months and then beyond that, even to this day. That can be frustrating for people around me if I get irritated with them. It’s almost like I’m menopausal…That’s been a challenge I guess.

The second code, in its last iteration, changed from redefining barriers and facilitators of resilience to the obstacle is the way to posttraumatic growth, which means that inside every obstacle is a chance to improve one’s condition. The transcripts revealed Robert’s choice to take something difficult and change his thoughts and behaviors as inspiration. This is directly seen in Interview 3, coded trauma behavioral change:

Trauma in the past has been responsible for behavioral changes that made for a much more promising life, like my addiction to alcohol for instance. The path that I took was so beneficial to me, and so it was like a gift.

Following this life experience, Robert gained access to spirituality that aided in his coping. In Interview 1, coded as growing spirituality, he stated, “As I got into the 12-step program with AA, I saw people…relying on Jesus and God to help ‘take the wheel’ and ‘show me the way.’ … My whole spiritual practice now is I’ve got immense respect for it.” In Interview 2, coded as embracing is the only way out, he stated, “We’ve got to embrace this [spirituality] because there’s only one way out.”

Robert’s brain was injured, so his mind adapted and converted to its purposes the obstacle to his acting. The Stoic Marcus Aurelius stated, “The impediment to action advances action” [20] (p. 59). What stood in Robert’s way became his way. This category is supported by analytic frameworks of Stoicism, which view challenges as opportunities, and the only thing in an individual’s control is response. The posttraumatic growth framework, with closer personal relationships and spiritual development, is also exhibited here. In the final interview with Robert’s wife, she too exhibited the obstacle is the way. She shared their interpersonal challenges with his poststroke anger. In the same passage, she also stated it is because of the stroke they are closer to this day. In Interview 4, she said,

Just like instantly he takes my head off. The first few times it happened, that just pissed me off…but I think we’re just a little bit closer. We’ve always had a really great relationship, but when you go through something like that sometimes it draws you closer.

Here they both demonstrate a greater appreciation of life and of valuing their relationship. She shared that they had been together for 17 years and finally decided to get married a year after the stroke.

This idea of an obstacle leading to positive change is found throughout examples in Robert’s life and all interview transcripts. Here, we gain insight into his perception, which is the first step to looking at a challenge and searching for ways to improve a problematic situation, even if only by your thoughts. This also is supported by the posttraumatic growth framework. In Interview 3, Robert stated,

Well, so I looked at the stroke, and I said early on I said, “I’ve got to find something here that’s good. I’m going to look back on years from now and say that that was the best thing that happened to me…if you’re going to be pulled through that misery it might as well be worth something.”

Due to the obstacle of having a stroke, Robert felt more ready to try something new to benefit himself; this is supported by the posttraumatic growth framework of embracing new possibilities. Also in Interview 3, he stated,

I think that the mindfulness deal, I was primed for it. I think that the people that suffered from a stroke are probably more likely to latch on to something like this. You’ve been through a trauma and if you know enough about what meditation can do if you stick with it. There’s a big reason to take it more seriously than you would have normally.

The factors that were labeled barriers became the exercises to propel his recovery further. One could quickly see the value of both the barriers and exercises to overcome them by detaching from judgment on their value as helpful or not. Robert acknowledged the stroke led to fear. By recognizing this previously described barrier to resilience, what mattered was his choice after labeling. He exhibits pushing and challenging himself, demonstrating excellent psychological resilience:

[What] really made me nervous was talking with clients over the phone in a compromised state. I was like, “OK well I’m shying away from this so let’s dive in.” So, the phone would ring, and I picked up the phone, and I started answering the phone and sometimes just saying, “Good morning, [business name].”

He shared without hesitation in Interview 2, “Yeah and so that’s why I started to do things like that, the things that I would shy away from. I just said, ‘OK, then this is what you need to do even if you don’t want to do it.’” He offered how he manages situations when the stroke impedes how he wants things to go. By maintaining openness and accepting the situation at hand (part of the posttraumatic growth domains of personal strength and vulnerability as well as closer relationships), he could ease and manage a stressful situation:

I always felt that I could tell people what was going on. That was my safeguard if I started to stumble with my words which was my biggest fear…it was funny because we had a serious meeting in [city, state], at the church. We had a quality problem that we needed to address with the architect and the general contractor. I needed to be involved in that and that was probably 3 months poststroke. I flew out there to have this serious meeting about it. It was a big financial consequence if we didn’t get things going our way and that took a lot of effort. I just came right out in the meeting, and said, “Hey, bear with me if I’m struggling with words because I had a stroke 3 months ago.” Everyone instead of being adversarial on an issue that they’re feeling for ya.

In review of the transcripts with new analytic frameworks, Robert exhibits the components of the domains defined in posttraumatic growth and Stoic philosophy. He exudes personal strength in his approach to accepting and responding to the challenges of having a stroke. He chooses not to withdraw, as seen with his best friend’s mother’s story of stroke, exhibiting both courage and discipline with daily rehab, meditation, and physical activity and jumping right back into work challenges. He demonstrates a greater appreciation of life by gratitude and mindfulness. He takes part in a new possibility by taking the mindfulness-based program, as reflected in Interview 2, and develops a deeper appreciation for spiritual growth.

### 3.4. Category 3: Poststroke Resilience: Mindfulness Embodied

The third category was named poststroke resilience: mindfulness embodied. Whereas Category 2 emphasizes how Robert responded to adversity by reframing obstacles and engaging in growth, Category 3 focuses more specifically on the embodied and ongoing role of mindfulness in sustaining poststroke resilience. The consequences are poststroke resilience due to the practice of mindfulness building from the substrates and environment for responding to the stressful event of stroke. In Interview 1, setting the stage for poststroke resilience, Robert described how he managed his physical, mental, and spiritual health, including his detailed plan for dealing with stressful events:

In a stressful period, there are three things that I focus on, and they help me get through. One is physical activity. The other is diet, and then the third is sleep. And if I can do well in all those three categories, [I can] pretty much tackle anything.

He also shared many stressful periods he has navigated, including running a business, raising two daughters, experiencing a divorce, having reduced time with his children, alcoholism, experiencing a potentially life-threatening illness (a pulmonary embolism), and selling his business.

Due to mindfulness, Robert also shared that he is now aware of not only his positive traits but his negative ones—and their potential effect on other people. When Robert referred to “the mindfulness deal” and his ongoing meditation practice in Interview 3, he was describing his engagement with the specific MBRfS curriculum outlined in the Methods, including daily sitting meditation, adapted mindful movement, and the use of mindful awareness to respond to stroke-related fears, anger, and uncertainty. In Interview 3, he stated, “Traits that made me successful in the past are not the best traits to live a peaceful life…my mind wanders and what kind of effect…that has on my ability to communicate with others.” Embodiment of mindfulness is a prominent factor in the ancient philosophy of Stoicism and a solid pathway to building psychological resilience. In Interview 3, Robert stated,

I’ve learned through mindfulness that my brain is always thinking about the future, and I spend very little time in the now…if I’m somewhere else and [wife’s name]’s talking, that’s not fair to her and anyone else that’s in my life…mindfulness I think is it is opened my mind to change.

This demonstrates the virtue of justice, meaning just behavior or treatment.

Part of Robert’s ability to reflect and grow since the stroke is evidenced in his ability to embody mindfulness. This is a key part of why he could express much of what is positive in his life following completion of the mindfulness-based group. In Interview 3, he stated,

I would say that reflecting back on it now, we’re coming up on 2 years in January. My dedication to meditation is the one thing that I can count on that is a result of my stroke. I think that it’s going to be responsible for a much better me…. I’ve learned a lot about myself through my meditation.

Several months following the program, in Interview 3, Robert asserted the value of the 8-week mindfulness-based program. Coded as a key element to stroke recovery, he stated, “What you have is a key element to stroke recovery that is currently is being ignored or unrealized.”

He shared further evidence of his ability to adapt in the face of adversity, wisdom, acceptance, and embodiment of mindfulness. Robert approached the situation of recovery from stroke with his response in the present moment. He avoided trying to change things as they are, or nonstriving, which is ultimately poststroke resilience. He shared in Interview 2,

I don’t think I have a goal of 100% recovery now. That was the initial goal, I’m going to recover. I mean who’s to know what is 100% recovery? And I think I’m probably better off rather than chasing some elusive thought and just embracing the process.

When we considered the longitudinal interviews together, the indices of mindfulness in Robert’s discourse appeared to shift over time. In Interview 1, he mainly described structured coping routines (such as exercise, diet, and sleep) and a strong drive for control and achievement, with relatively few explicit references to observing thoughts or accepting limits. By Interview 2, his language more often reflected acceptance of the stroke (“We’ve got to embrace this because there’s only one way out”) and deliberate exposure to feared situations (e.g., answering the phone or returning to high-stakes meetings), suggesting emerging mindful courage and nonavoidance. In Interview 3, he repeatedly articulated observing his own thinking patterns (“My brain is always thinking about the future”), noticing their impact on relationships, and cultivating non-striving (“Rather than chasing some elusive thought and just embracing the process”), which we interpret as stronger indices of embodied mindfulness in daily life.

We present these shifts narratively rather than numerically, because our aim was to illuminate the evolving role of mindfulness in Robert’s recovery rather than to quantify code frequencies in a statistical sense.

### 3.5. Carepartner Interview

Robert’s carepartner, his wife, acknowledged the benefit Robert experienced during his time with the pilot study in the fourth interview: “Thank you to both of you because he’s gotten so much from participating in your study and just being associated with you, so thank you.”

During her interview she triangulated many aspects of the first three interviews, such as this example of courage:

He’s been incredibly brave in his recovery…. We would get to the point where we go through the drive-thru and he would order. He would attempt to do the ordering, and he would stumble over it, and he would keep doing it. Instead of making me holler across.

Robert’s partner reminded the nurse clinician of the duty of a nurse to the patient, the environment, and the people surrounding the patient. She acknowledged it would have been helpful if the discharge education from the hospital or clinic had introduced the concept of poststroke anger or other emotional issues that are common in recovery from stroke. Here, she offered another opportunity for healthcare providers to better equip those who care for people who have experienced stroke. In Interview 4, she stated,

I think it would be nice…if somebody would have said, “Here’s what you might be able to expect….” Maybe the short fuse thing…I’m thinking back to a conversation that he and I had. I think maybe he might have brought it up in one of the study groups, but some of that he did with you guys a long time ago…he said some of the other people were having the same issue. Which actually made me feel a little bit better, but I think it would have been nice to know that.

She commented that because Robert recovered functionally so quickly, she did not need additional social and emotional support for his difficulties with aphasia, beyond her daughter and sister: “My daughter, who’s my best friend…I talked to my sister, who’s another best friend…my parents just love Robert, so they were there.” She reflected further on her personal low needs for support: “I didn’t…I’m not sure why…except that he showed improvement so quickly.”

She utilized journaling as a coping mechanism both before and after the stroke, including when she went through a divorce, as shown in Interview 4: “Learning about other ways to kind of deal with things and approach the world and so I started journaling, and I’ve always liked to write…I have a very strong journaling practice.” In Stoicism, journaling is a reflective, purposeful practice that helps a person cultivate a deep understanding of their thoughts, feelings, and actions. The literature also suggests journaling helps improve well-being after traumatic and stressful events [23].

When describing her relationship to Robert within the context of stroke recovery, she used the term “caregiver” early on in the interview but later clarified she did not feel this term applied to her. At first, she said,

I just don’t know. I guess if there was a support person or some information…From a caregiver standpoint, that kind of stuff would be really helpful…. I don’t recall any talking to me about what it could mean for me as the caregiver, nobody addressed that.

Later, she clarified,

The term “caregiver” doesn’t exactly fit because he hasn’t really needed that much care per se…. He’s been able to take care of himself, so the term “caregiver” for me is a little bit different feeling if that makes sense. And I could see how again if he had you know he couldn’t walk or he couldn’t use an arm, or his speech hadn’t come back, or say you know, I think that role would have ended up being much bigger.

Robert’s wife is not alone in feeling that “caregiver” is not the right fit to describe her role. Other stroke survivors have embraced the term “carepartner” rather than “caregiver” to reflect that stroke affects the whole family, not just the person who experienced the stroke. Furthermore, this term emphasizes the collaborative nature of holistic stroke rehabilitation in which all members of the family, including the stroke survivor, play roles in recovery [24,25].

The carepartner interview also demonstrated that in this case, Robert’s wife was one step removed from the stroke. In the posttraumatic growth framework, perhaps her life was not changed enough for her to feel motivated to pursue new things, such as seeking out mindfulness or additional support for Robert’s aphasia, given its rapid improvement. She did not experience much disruption in her usual activities, responding to the question about that with, “I don’t think so. We both feel very blessed.” In responding to inquiry regarding changing energy levels, she stated, “Nothing, I don’t think so.” Regarding changes in her job, she stated, “I don’t really recall that I did right now, my client load is light enough that I can just work around it.” She reported only mild social changes for a brief time:

For a while it had an impact on our social life because he wasn’t comfortable going out. Because he couldn’t communicate with people. It took him a little bit to feel comfortable doing things like that. That’s totally fine with me; I wasn’t going to push it.

To assess the degree to which the mindfulness-based training program from the pilot study aided Robert and his partner in his recovery from stroke, holistically as a couple, it is best to focus primarily on the stroke survivor. Robert’s earnest nature is shown in his self-reported bi-daily meditation practice, regarding which he stated, “Be taken by a stroke. So that is what keeps me in my seat in the mornings for 20 min or 10 min before I go to bed.” He also noted the potential meditation has to produce change: “I think meditation is the way I can make that change.” Robert’s wife earnestly supported meditation and had tried it with success before herself, but she was not currently practicing or pursuing practicing. She shared,

[In the past I have] dabbled with yoga and meditation. I love yoga. I haven’t done much of it at all lately. I’ve had periods before…I’ve also dabbled with meditation. I love it. I believe in it, but I do not practice. It’s on the list of things that would be really good for me to do but I have not been [practicing] lately.

It appears that Robert’s partner had enough support, and because she was one step removed from the stroke, the challenges of stroke recovery did not significantly impact her needs or motivations to make changes in her life, such as in her case returning to the practice of mindfulness meditation.

### 3.6. Selective Code: Sand Mandala, the Essence of Resilience, Growth, and Thriving After Stroke

In reviewing the four transcripts and reflecting on our experiences with Robert from prior interviews and focus groups of the pilot study, we developed a selective code as we continued to integrate and refine our theory. All the categories and reviews of Robert’s personal artistic website led to the potential conceptualization of the sand mandala. A sand mandala is inherently ephemeral art created from grains of colored sand; it is a metaphor for the universe and regarded as something sacred in Tibetan Buddhism [26]. We used the sand mandala as an integrative selective code because repeated elements in the interviews—impermanence, reconstruction, disciplined practice, transformation through disruption, and carrying forward what remains after loss—were consistently reflected across Robert’s narrative and self-understanding. We therefore present the sand mandala as an interpretive synthesis of recurrent empirical patterns in the case rather than as a metaphor imposed independently of the data.

Robert compared his chalk art, featured on his personal website, to a sand mandala:

I remember the first time that I saw a group of Buddhist monks make a sand mandala. They spent 2 days laboring to create this amazingly colorful work of art only to destroy it. This stark example of the transitory nature of life as exemplified by their work had a profound effect upon me. I find that chalk art serves the same purpose. Chalk is an outstanding medium for creating art and allows the artist the ability to produce a masterpiece in a similar way to using pastels. Different colors and hues can be attained by mixing different colors and blending them together to achieve a precision found in a professional studio. The fact that the resulting artwork will only last until the next rain actually enhances the beauty of the effort.

Sand mandalas can be created for various reasons, including to heal communities and the environment [26]. They function as a contemplative aid in visualization meditation for the artists and those observing or praying during their creation. They are made from ground marble and put into bright colors of sand. Each grain of sand will give one blessing. This form of Buddhism is about developing your mind’s potential, a way of transforming the mind. This creation demonstrates a very important Buddhist belief in impermanence. When humans are transformed by death, their essence remains and carries on. A second important belief is nonattachment, as shown in the idea of the destruction of the sand mandala soon after its creation; the sand mandala is returned to the elements.

Initially, we referred to this selective code as sand mandala, making it through the components of resilience. Robert listed several challenging circumstances leading to his stroke and shared aspects of himself that were incredibly resilient before our interaction in the mindfulness-based pilot study, including the category of antecedents to poststroke resilience: personality, values, behavioral modeling, and environment. However, we decided against the wording “making it through.” For many, if not most, stroke survivors, stroke recovery is nonlinear, and recovery is a lifelong process. “Making it through” is not quite the right description because “making it through” implies an end or something static.

Instead, we returned to the data and found embodied mindfulness. During both the pilot study, 10 months prior, and the present case study, Robert gave several examples of embodied mindfulness, that is, increasing awareness of himself and his actions. He focused on what the Stoic philosopher Epictetus [22] said is the most efficacious gift, the essence of human nature: the faculty of choice. The core idea in Stoicism is we do not control what happens to us in life, but we control how we respond. Robert described it as being “new at the game.” This is evidenced beginning in Interview 3, in which he stated,

That is what the mindfulness has done for me…it’s made me aware that my mind takes me to places that don’t benefit me whatsoever, in fact they harm me and you know so I see that now where I didn’t see that before you know before I would recognize that I would get upset with traffic when I was under a lot of stress and I’d say, “OK you need to dial it back a little bit.”… Now that I have just finally realized that you know how aberrant that behavior is so I’ve got some learning to do and I’m just, I’m new at the game.

Mindfulness permitted Robert a better insight into his behaviors and reactions and the ability to stop and choose. He additionally referenced the perceived benefit of recovery from stroke as the betterment of himself. This reflection led to the active, selective code of sand mandala, the essence of resilience, growth, and thriving after stroke. Robert referenced imagining himself 10 to 30 years from now, growing with his newly found meditation skills in Interview 3:

Ten years from now, I am going to go, “Boy was that the best thing to happen to me…Be taken by a stroke.” …You shared a great story in our class about Mohini the tiger. How he is still walking the same path even though he’s got a whole new environment to him. That is how I see myself is that I’ve got another 30 years and what am I going to do with those? Am I going to walk this path that I’ve walked for many years. Or how do I change? I think meditation is the way I can make that change. I don’t see anything else that can have the impact that meditation could have on me from being a Mohini. That is what drives is that belief that there is something better than I have right now.

## 4. Discussion

### 4.1. Implications for Holistic Stroke Rehabilitation Practice

The implications of this study are that mindfulness-based interventions helped foster and strengthen psychological resilience in this stroke survivor, which in turn offers clear objectives for long-term, whole-person stroke rehabilitation. Although researchers have identified mindfulness as something that promotes psychological resilience in stroke recovery, it is not yet known what the best way is to encourage survivors to rapidly accept a traumatic event like stroke and what will impede progress. This case study serves as an example of the effectiveness of mindfulness-based interventions on one individual’s extreme psychological resilience while insulated by adequate social determinants of health and the cushion of social structural stability. It also demonstrates the importance of a flexible mindset to adapting to a variety of lifelong challenges. The posttraumatic growth mindset and the principles of Stoicism that this participant revealed to us throughout his remarkable recovery from stroke were ingrained through a lifetime of engagement with personal and interpersonal experiences. As with any trauma, stroke can lead to a variety of immediate and delayed emotional, physical, cognitive, behavioral, and existential changes [27]. This case serves as a practical example of how mindfulness practiced within a supportive community of stroke survivors fosters personal growth, nurtures hope for the future, and strengthens resilience throughout the lifetime of recovery.

These observations are consistent with prior work suggesting that psychological resilience and mindfulness may be associated with improved emotional adjustment and quality of life after stroke, but this case contributes a more process-oriented account of how those dynamics may unfold in one individual’s daily life over time. In contrast to outcome-focused quantitative studies, the present case highlights the interaction of prestroke traits, social and environmental supports, responses to adversity, and sustained mindfulness practice within a single long-term recovery trajectory.

Future investigations could build on this case in several ways. Prospective, mixed methods studies of mindfulness-based interventions for stroke survivors could combine in-depth qualitative interviews with validated quantitative measures of resilience, mindfulness, mood, and quality of life in larger and more diverse samples. Longitudinal designs with assessments before, during, and after the intervention would allow researchers to examine how resilience-related narratives and mindfulness indices evolve over time and how these trajectories relate to functional, emotional, and social outcomes. Comparative work could also explore how social determinants of health and carepartner involvement shape who is most likely to benefit from programs like Mindfulness-Based Recovery from Stroke and under what conditions these interventions are feasible and acceptable in routine stroke rehabilitation settings. These implications should be interpreted cautiously, as they derive from a single instrumental case and are intended to be exploratory rather than prescriptive.

### 4.2. Limitations and Quality Standards

In this inquiry we sought to generate a particular understanding only of the complexity of the case—Robert, an extreme in terms of psychological resilience. This case study has several important limitations. First, it presents an in-depth analysis of a single stroke survivor with exceptional psychological resilience and one carepartner, and the findings are not intended to be statistically generalizable to all people living with stroke or to all recipients of mindfulness-based interventions. It is possible that Robert represents an extreme or atypical trajectory, given his pre-existing personality traits, life history, and access to psychosocial and material resources, including socioeconomic stability and ready access to healthcare and community resources.

This case was also intentionally selected as a case of high psychological resilience, which introduces selection bias and limits transferability to stroke survivors with different personal, social, or structural circumstances. In addition, because members of the research team were involved in the pilot intervention and related clinical context, there is potential for interpretive bias despite efforts at reflexivity, triangulation, and team-based analysis.

Second, without a comparison or control group and without experimental control over extraneous variables, we cannot determine the unique causal contribution of the Mindfulness-Based Recovery from Stroke program versus other influences (e.g., natural recovery, ongoing rehabilitation, family and peer support, or preexisting spiritual and recovery practices) on his psychological resilience and posttraumatic growth. This study should therefore be understood as hypothesis-generating rather than as providing definitive evidence of intervention efficacy.

Third, our analysis relies on self-report and researcher interpretation, which are vulnerable to recall bias, social desirability, and our own theoretical commitments, despite our efforts to maintain reflexivity and triangulate Robert’s accounts with the carepartner interview and other artifacts. Finally, because this is an in-depth, instrumental single case, we did not apply formal quantitative frequency analysis or standardized quantitative measures of resilience or mindfulness, as such approaches could suggest a level of measurement precision and generalizability that exceeds the scope and intent of this study.

Trustworthiness is ensured by multiple sources of data and triangulation with the participant’s wife and carepartner. We plan to share this work with other participants to ensure the validity of the findings.

## 5. Conclusions

Robert was equipped with many of the seemingly optimal components of the society-to-cells resilience theory of resilience on all six levels. At the cellular level, he had foundations of good health related to nutrition, sleep, and environment, despite experiencing cellular death and pathophysiological changes of stroke. At the individual level, he had adequate esteem, outlook, and agency. He was supported by family and community and had an optimal place in society despite the threat of life transitions with changes in his career. Additionally, he demonstrated his ability to respond to trauma and to transform himself by embracing the principles of Stoicism. Before encountering the mindfulness-based program, he exhibited the belief that the obstacle is the way, the ability to view the obstacle as the path to growth. Because the stroke was traumatic enough, a cascade of events led to posttraumatic growth in part by opening him to try something new. The mindfulness-based pilot program reinforced many aspects of resilience within him. It also gave him something new: an embodiment of mindfulness, greater access to his thoughts, a like-minded community, reduced isolation, determination of action, and the essence of human nature: the faculty of choice. By Stoic principles, he navigates his life with the virtues of discipline, courage, wisdom, and justice, revealing exceptional resilience. This bounded case study explored the benefit of the mindfulness-based pilot study as embodied mindfulness of the primary participant. This exploration revealed the evidence of mindfulness action of the carepartner’s strong journaling practice predating the stroke.

The sand mandala is considered a perfect universe, and after its laborious creation, it is destroyed, representing the impermanence of life. The components, however, are there to create another mandala; the sand, like the elements of resilience, remains. The meditation of the sand mandala represents our case study participant’s ability to keep going, change, grow, and cope, and yet carry with him what he needs to not only make it through the aftermath of the stroke but thrive. For now, the analytics pause on the selective code of sand mandala, the essence of resilience as one aspect of life after stroke.

This instrumental case study provides an exploratory account of how resilience, adversity, and mindfulness may interact in one highly resilient stroke survivor’s long-term recovery. The findings suggest that prestroke personal and environmental strengths, the active reframing of obstacles, and the embodied practice of mindfulness may have worked together in shaping this participant’s poststroke adaptation. Because this is a single, extreme case, these findings are not generalizable and should be understood as hypothesis-generating. Future research should examine these processes in larger and more diverse stroke populations, using longitudinal and mixed methods designs to better understand how mindfulness, resilience, and social context interact over time.

## Data Availability

Data are available on request from the authors.

## Abbreviations

The following abbreviations are used in this manuscript:

AA	Alcoholics Anonymous
IRB	Institutional Review Board
